# Genome-wide identification and expression analysis of GRAS transcription factors under cold stress in diploid and triploid *Eucalyptus*

**DOI:** 10.3389/fgene.2025.1436285

**Published:** 2025-01-29

**Authors:** Jiannan Liu, Hao Chen, Chenhe Li, Kang Du, Jun Yang

**Affiliations:** State Key Laboratory of Tree Genetics and Breeding, National Engineering Research Center of Tree Breeding and Ecological Restoration, Key Laboratory of Genetics and Breeding in Forest Trees and Ornamental Plants of Ministry of Education, College of Biological Sciences and Technology, Beijing Forestry University, Beijing, China

**Keywords:** GRAS, genome-wide analysis, cold stress, *Eucalyptus*, triploid

## Abstract

The GRAS [GRI (Gibberellic Acid Insensitive), RGA (Repressor of GAI-3 mutant), and SCR (Scarecrow)] transcription factors play a pivotal role in the development and stress responses of plants. *Eucalyptus* is an important fast-growing tree species worldwide, yet its poor cold tolerance limits its cultivation range. This study conducted a bioinformatics analysis of *Eucalyptus* grandis *GRAS* family and investigated the expression patterns of GRAS genes in different ploidy *Eucalyptus* under cold treatment. This study identified 92 *EgrGRAS* genes, which were divided into eight subfamilies. Interspecies synteny analysis found that *E. grandis* and Populus trichocarpa have more syntenic *GRAS* gene pairs. Chromosome localization analysis revealed that 90 *EgrGRAS* genes were found to be unevenly distributed across 11 chromosomes. Gene structure analysis found similar intron-exon structures in *EgrGRAS* genes. Protein motif analysis revealed that proteins within the same subfamily have certain structural similarities. The physical and chemical properties of the proteins encoded by *EgrGRAS* genes vary, but the ranges of amino acid numbers, molecular weights, and isoelectric points (pI) are similar to those of *GRAS* proteins from other species. Subcellular localization prediction using software found that 56 members of *EgrGRAS* family are localized in the nucleus, with a few members localized in the cytoplasm, chloroplasts, and mitochondria. Tobacco subcellular localization experiments verified a nuclear-localized *GRAS* transcription factor. *Cis*-acting element analysis predicted that *EgrGRAS* genes are involved in the growth as well as the response to hormones, light induction, and low-temperature stress. Transcriptome data analysis and quantitative real-time PCR (qRT-PCR) experiments in diploid and triploid Eucalyptus urophylla found that some EgrGRAS genes exhibited upregulated expression under different cold treatment durations, with certain genes from the LISCL, PAT1, and DELLA subfamilies significantly upregulated in triploid Eucalyptus. These *EgrGRAS* transcription factors may play an important role in *Eucalyptus* response to cold stress. The study lays a molecular foundation for the breeding of cold-resistant *Eucalyptus* varieties.

## 1 Introduction

The GRAS transcription factor family is named after the initial discovery of three family members: Gibberellic Acid Insensitive (GAI), Repressor of GAI-3 mutant (RGA), and Scarecrow (SCR) (Di Laurenzio et al., 1996; Peng et al., 1997; Pysh et al., 1999; Silverstone et al., 1998). GRAS transcription factors are plant-specific regulatory proteins whose structure and function have been extensively studied. These proteins typically consist of 400–770 amino acid residues and possess a conserved *C*-terminal domain. This domain contains several characteristic polypeptide motifs, including leucine-rich region I (LHRI), leucine-rich region II (LHRII), VHIID, PFYRE, and SAW motifs (Bolle, 2004). VHIID represents the core region of GRAS transcription factors, featuring a highly conserved P-N-H-D-Q-L structural unit with an L-R-I-T-G terminal structure (Tian et al., 2004). LHRI and LHRII flank VHIID on both sides. PFYRE consists of three pairs of conserved protein sequences P, FY, and RE, while SAW consists of three conserved amino acids R-E, W-G, and W-W (Lee et al., 2008). Compared with the conserved *C*-terminal, the *N*-terminal of GRAS family proteins are more variable and contain many inherently disordered domains (IDRs). *N*-terminal is a functional component of GRAS family proteins (Sun et al., 2012), which can recognize and bind to different specific proteins and play different roles in plants.

At present, members of *GRAS* gene family have been identified in a variety of plants, such as 54 members in tomato (*Solanum lycopersicum*) (Niu et al., 2017), 32 in Arabidopsis (*Arabidopsis thaliana*) (Tian et al., 2004), 60 in rice (*Oryza sativa*) and 106 in Populus (*P*. *trichocarpa*) (Liu and Widmer, 2014). Among them, *GRAS* gene family in Arabidopsis and rice can be divided into nine subfamilies (Hirsch and Oldroyd, 2009). The SCR and SHR proteins are involved in the regulation of root growth in Arabidopsis (Helariutta et al., 2000), while the MOC1 protein participates in the establishment and maintenance of leaf axil in rice (Lin et al., 2012). The PAT1 protein in Arabidopsis is involved in the signal transduction of phytochrome A (phyA) (Bolle et al., 2000), and the SLR1 protein in rice inhibits the signal transduction of gibberellin (Hirano et al., 2010). In recent years, an increasing number of studies have indicated that GRAS transcription factors not only play a significant role in plant growth and development, signal transduction, and other aspects, but also have substantial functions in response to biotic and abiotic stress responses in plants. Under biotic stress, OsCIGR2 in rice reduced the number of cell deaths by inhibiting the invasion of pathogenic bacteria (Tanabe et al., 2016); under various environmental stresses, NtGRAS1 in tobacco (*Nicotiana tabacum*) regulated stress resistance by increasing the level of reactive oxygen species (Czikkel and Maxwell, 2007); under high salinity and drought stress, the expression of *Gh*_*A0IG0682* and *Gh*_*A04G0081* in upland cotton (*Gossypium hirsutum* L.) was upregulated (Zhang et al., 2018). Specifically, some studies have indicated that GRAS transcription factors are associated with the plant response to cold stress. Low temperatures affected the expression of *GRAS* genes in bananas (Tong et al., 2023). VaPAT1 played a critical role in the low-temperature stress response of grape calli by regulating jasmonic acid (JA) biosynthesis (Wang et al., 2021). DELLAs were involved in the CBF1-induced chilling tolerance mechanism, enhancing the freezing resistance of Arabidopsis (Achard et al., 2008).

*Eucalyptus* is one of the most widely cultivated tree species in the world, known for its rapid growth rate, high-quality timber, and strong carbon sequestration capabilities. It is commonly used in applications such as timber production, papermaking, and board manufacturing (Vilasboa et al., 2019). Originally native to Australia, *Eucalyptus* has been extensively introduced and cultivated in China since the mid-1990s (Zhang and Wang, 2021), where it is grown on a large scale in Guangxi, Guangdong, Sichuan, and Hainan as a fast-growing and high-yielding forest tree species. With the continuous development of China’s *Eucalyptus* industry, the cultivation range of *Eucalyptus* has gradually moved northward. However, its insufficient cold tolerance has limited its widespread promotion. Studies have indicated that plants undergoing natural or artificial polyploidization tend to exhibit enhanced tolerance to various stresses, which positively impacts their growth and results in a broader environmental adaptability (Bon et al., 2020; Brochmann et al., 2004; Song et al., 2020). *Eucalyptus* does not possess natural polyploids; however, the artificial induction of chromosome doubling in megaspores to obtain triploidy was successfully achieved (Yang et al., 2018). This makes it possible to study the expression response of *GRAS* gene family members in *Eucalyptus* at different ploidy levels under cold stress.

To explore the expression differences of *GRAS* gene family members under cold stress in triploid and diploid *Eucalyptus*, this study first identified the members of *EgrGRAS* gene family at the whole-genome level. Then, their physicochemical properties, subcellular localization, evolutionary relationships, sequence characteristics, chromosomal distribution, synteny, and *cis*-acting elements were analyzed. Finally, using transcriptome sequencing data and qRT-PCR experiments, the expression patterns of *GRAS* genes in triploid and diploid *E. urophylla* under cold stress were analyzed, uncovering *EgrGRAS* family members that significantly respond to cold stress in triploid *Eucalyptus*. This study lays the foundation for further exploration of *Eucalyptus* cold resistance gene resources and the molecular mechanisms underlying the response of triploid *Eucalyptus* to cold stress.

## 2 Materials and methods

### 2.1 Identification of EgrGRAS family members

Identification of *Eucalyptus grandis* GRAS family members was conducted using two approaches: (1) The whole-genome information and annotation files of *E. grandis* (v2.0) were downloaded from Phytozome (https://phytozome-next.jgi.doe.gov/). Arabidopsis *GRAS* genomic data were obtained from TAIR (https://www.arabidopsis.org/) (Lamesch et al., 2012). Utilizing the TBtools software (Chen et al., 2020), BLAST P alignment searches were performed to identify members of EgrGRAS family (E-value ≤ 1e^−5^), using the amino acid sequences of 32 ATGRAS members as the query sequences. (2) Hidden Markov Model (HMM) profile files were downloaded from InterPro (https://www.ebi.ac.uk/interpro/) (Blum et al., 2021). The Simple HMM Search program within the TBtools software was employed, in conjunction with the conserved GRAS domain (PF03514) to search (E-value ≤ 1e^−10^) for EgrGRAS sequence data. By comparing the analytical results of the two methods, we preliminarily ascertain the members of the EgrGRAS family. To confirm that the predicted proteins contain GRAS domain, we utilized NCBI-CDD (https://www.ncbi.nlm.nih.gov/Structure/bwrpsb/bwrpsb.cgi) (Lu et al., 2020) and SMART (http://smart.embl-heidelberg.de/) (Letunic et al., 2021) for their validation and assessment. Proteins lacking a complete GRAS domain were excluded, thereby determining the final members of EgrGRAS transcription factors.

The Protein Parameter Calc program within the TBtools software was utilized to predict the amino acid count, molecular weight, isoelectric point, and other physicochemical properties of EgrGRAS members. Additionally, WoLF PSORT (https://wolfpsort.hgc.jp/) was employed for the prediction of subcellular localization of these proteins. Meanwhile, we randomly selected an *EgrGRAS* to carry out the subcellular localization experiment. The *Eucgr.H01257.1.v2.0* (EgrGRAS60 in Supplementary Table S1) were isolated from the cDNA of *E*. *urophylla*. The coding sequence (CDS) of EgrGRAS60 without a stop codon was integrated into the pCAMBIA1300-eGFP vector. The primers are listed in Supplementary Table S2. The constructed fusion expression vector was transformed into *Agrobacterium* GV3101 (pSoup-p19). Expand and resuspend the positive clones screened by PCR. The bacterial suspension was injected into the lower epidermis of *Nicotiana benthamiana* leaves when the OD_600_ value was 0.5 - 0.8. The infected tobacco plants were cultivated for 36 - 48 h. Subsequently, the fluorescent signal of the tobacco leaves was observed by using a laser confocal microscope (Leica TCS SP8; Leica, Wetzlar, Germany).

### 2.2 Phylogenetic analysis of GRAS members

The amino acid sequences of EgrGRAS family were subjected to multiple sequence alignment using the Clustal W tool available in MEGA 11 (Tamura et al., 2021). Trim the results using the Quick Run TrimAL program in TBtools software. Subsequently, a phylogenetic tree of EgrGRAS was constructed employing the neighbor joining (NJ) method in MEGA 11, with default parameters and 1,000 bootstrap replicates. Preliminary grouping of EgrGRAS family members was conducted. The iTOL software was used to beautify the phylogenetic tree (https://itol.embl.de/) (Letunic and Bork, 2024).

*GRAS* genes in Populus have been previously reported (Liu and Widmer, 2014). Utilizing protein sequences retrieved from the PlantTFDB database (https://planttfdb.gao-lab.org/index.php) (Jin et al., 2017), 102 PotriGRAS family members located on the chromosomes have been selected. These were then combined with 32 ATGRAS protein sequences, which had been previously obtained from TAIR, and 92 EgrGRAS protein sequences that had been identified, into a single file. The sequences were aligned using Muscle5 (https://www.drive5.com/muscle5/) (Edgar, 2004). Following alignment, the sequences were trimmed using the trimAl tool (https://github.com/inab/trimal) (Capella-Gutierrez et al., 2009). Subsequently, a phylogenetic tree for the three species was constructed using the Maximum Likelihood (ML) method with the best-fit model “JTT + F + R5” and 1000-bootstrap values in IQ-TREE (Minh et al., 2020). Finally, the evolutionary tree was visualized and embellished using iTOL.

### 2.3 Analysis of gene structure and conserved motif

The conserved motifs analysis of EgrGRAS proteins was conducted by MEME (https://meme-suite.org/meme/tools/meme) (Bailey et al., 2015), with the maximum number of motifs set to 10, and all other parameters were retained at their default values. The resulting files, along with the phylogenetic tree files and the GFF3 database obtained from Phytozome, were collectively imported into the Gene Structure View program within the TBtools software. This facilitated the generation of a comprehensive diagram depicting the gene structure of *EgrGRAS* and the conservation of protein motifs.

### 2.4 Chromosomal localization and synteny analysis

The gene location distribution of *EgrGRAS* gene family members across the 11 chromosomes was obtained using the Gene Location Visualize program within the TBtools software, by incorporating *E. grandis* GFF3 files and *GRAS* gene IDs. The genome data and the gene annotation files of *A*. *thaliana* (TAIR10), *O*. *sativa* (v7.0), *S*. *lycopersicum* (ITAG5.0), and *P, trichocarpa* (v3.1) were downloaded from Phytozome (https://www.arabidopsis.org/). The One Step MCScanX-Super Fast plugin (Wang et al., 2012) was installed within the TBtools software, which was subsequently utilized for synteny analysis between *E. grandis* and the other species (E-value ≤ 1e^−10^). Finally, the Dual Synteny Plot for MCscanX program in TBtools software was employed to generate the syntenic analyzing graphs.

### 2.5 The prediction of *cis*-element

The 2,000 base pairs upstream of the transcription start codon of *EgrGRAS* genes were extracted using the Gtf/Gff3 Sequences Extract and Fasta Extract programs within the TBtools software. Subsequently, the promoter sequences of *EgrGRAS* genes were subjected to *cis*-acting element prediction using PlantCARE (http://bioinformatics.psb.ugent.be/webtools/plantcare/html/) (Lescot et al., 2002). The data were filtered and the results were organized. Finally, the Simple BioSequence Viewer program within the TBtools software, in conjunction with the evolutionary tree files, was utilized to visualize the key *cis*-acting elements.

### 2.6 Transcriptome data analysis of *EgrGRAS* gene family members

We used previously reported transcriptome sequencing data to conduct expression analysis of *EgrGRAS* (Chen et al., 2021). These data were obtained from young leaves of diploid and triploid *E. urophylla* seedlings. Transcript abundance was represented as Fragments Per Kilobase of transcript per Million mapped reads (FPKM). LT1, LT2, LT3, and LT4 refer to cold treatment for 6, 12, 24, and 48 h, respectively. After 48 h of treatment at 4°C, the seedlings were returned to a 25°C environment for recovery: Re1 and Re2, indicating recovery periods of 12 and 24 h, respectively. The non-cold treatment group (CK) was used as a control. The expression levels of different transcripts of the same gene were summed. The data for the expression levels of *GRAS* genes were integrated separately for diploids and triploids under CK, LT1, LT2, LT3, LT4, Re1 and Re2, ensuring that the majority of the FPKM values exceeded 1 (Supplementary Tables S3, 4). Heatmaps of *GRAS* gene expression under different treatments for both diploid and triploid *E. urophylla* were generated using the HeatMap program in TBtools software, with Log_2_(FPKM+1) scale, normalizing rows and setting hierarchical clustering. To further explore the impact of differential expression of *GRAS* genes on the cold tolerance of leaves in *E. urophylla* with different ploidy levels, the Log_2_ (fold change) values of differentially expressed *GRAS* genes in the leaves of triploid and diploid *E. urophylla* under the aforementioned seven treatments were integrated (Supplementary Table S5). A heatmap of gene differential expression was constructed using TBtools software, with setting hierarchical clustering.

### 2.7 Cold treatments of *Eucalyptus urophylla* and qRT-PCR analysis

Diploid and triploid *E*. *urophylla* seedlings originated from the Guangxi Dongmen Forest Farm. After a period of domestication in the greenhouse at Beijing Forestry University, seedlings (approximately 3 months old) with consistent growth were selected for the experiment. The seedlings maintained at 25°C served as the control group. The experimental groups included treatments at 4°C for 6, 12, 24, and 48 h, as well as recovery at 25°C for 12 and 24 h following 48 h of treatment at 4°C, totaling six groups. All *E*. *urophylla* seedlings were grown under a 16/8 h (light/dark) photoperiod. Young leaves were collected after each treatment, with three biological replicates for each group. Samples were immediately frozen in liquid nitrogen and stored at −80°C.

The total RNA extraction was carried out using an RNAprep Pure Plant Plus Kit (Tiangen, Beijing, China, cat DP441). cDNA was quantitatively prepared with a cDNA Synthesis Kit (Tiangen, Beijing, China, cat KR106). The qRT-PCR was conducted on the 7,500 Fast instrument (Applied Biosystems, MA, United States) using 2 × Sybr Green qPCR Mix (Aidlab, Beijing, China, cat PC33). *Actin2* (*Eucgr.I00241*) was utilized as a reference gene (Cassan-Wang et al., 2012). The qRT-PCR primers were obtained from qPrimerDB (https://qprimerdb.biodb.org/), and the primers of the seven analyzed genes are presented in Supplementary Table S6. The relative expression levels of the target genes were calculated using the 2^−ΔΔCT^ method (Livak and Schmittgen, 2001) in Microsoft Excel. GraphPad Prism version 9.5 (GraphPad Software, San Diego, California, www.graphpad.com) was employed for analysis of variance (ANOVA), multiple comparisons and plotting. *p < 0.05 and **p < 0.01.

## 3 Results

### 3.1 Identification of *EgrGRAS* genes and subcelllar localization analysis

Through BLAST P and HMM screening, as well as validation with the CDD database and SMART, a total of 92 members of *EgrGRAS* gene family were identified. The obtained sequence IDs were renamed from *EgrGRAS1* to *EgrGRAS92* (Supplementary Table S1). The basic characteristics and property of EgrGRAS family members were listed in Supplementary Table S1. The length of EgrGRAS proteins ranged from 267 amino acids (EgrGRAS82) to 817 amino acids (EgrGRAS74), with molecular weight spanning from 31,472.38 Da (EgrGRAS82) to 92,755.82 Da (EgrGRAS74). The pIof the proteins varied between 4.86 (EgrGRAS1) and 9.15 (EgrGRAS88). Judging from the pI value, most of EgrGRAS proteins (77/92) were acidic, and the rest EgrGRAS proteins (15/92) were alkaline. Except for EgrGRAS68, which had an average hydrophilicity index of 0.007, all other members were hydrophilic, with an index less than zero. The majority of EgrGRAS members were unstable, with instability index exceeding 40. Only EgrGRAS63 had an instability index of 37.38, indicating that it may be a stable hydrophilic protein. The higher the aliphatic index of a protein, the greater its thermal stability. For the EgrGRAS proteins, the aliphatic index ranged from 68.74 (EgrGRAS3) to 96.62 (EgrGRAS58).

**FIGURE 1:**
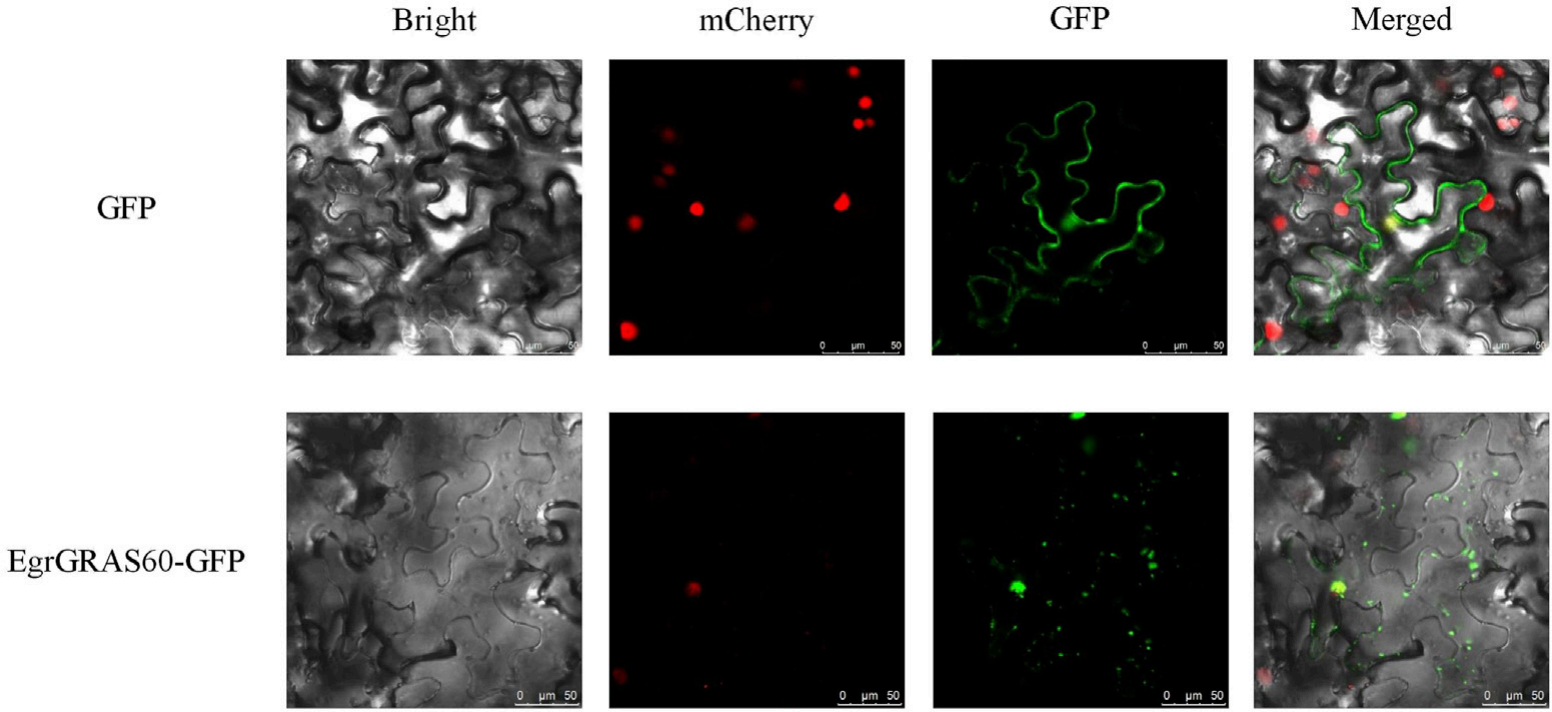
Subcellular localization of EgrGRAS60-GFP upon transient expression in tobacco leaves. GFP without fusion to EgrGRAS60 was also transiently expressed and served as a control. mCherry was used as a nuclear marker. Scale bar = 25 μm.

Subcellular localization prediction results indicated that 56 were localized to the nucleus, 19 to the cytoplasm, 16 to the chloroplasts, and one (EgrGRAS61) to the mitochondria among 92 EgrGRAS proteins. Additionally, we performed transient transformation of the cloned EgrGRAS60. In the control, the GFP protein in the empty vector was localized to the cell membrane and nucleus. Whereas, in the fusion expression vector EgrGRAS60-GFP, fluorescence was not present in the cell membrane but was prominently detected in the nucleus (Figure 1). This finding suggested that EgrGRAS60 was localized in the nucleus, which was in accordance with the prediction made by the WoLF PSORT software (Supplementary Table S1).

### 3.2 Phylogenetic relationships of EgrGRAS proteins

**FIGURE 2:**
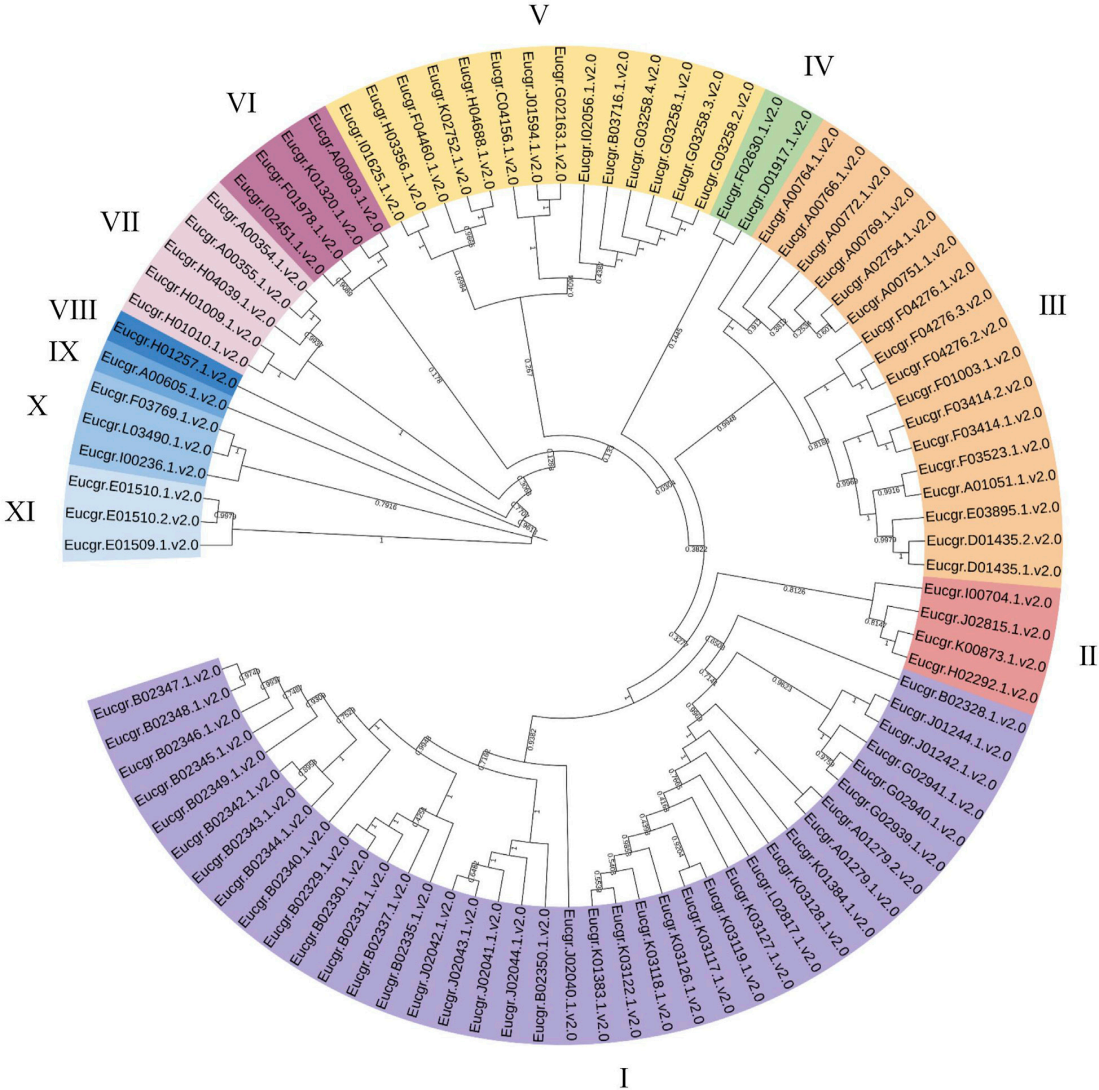
Phylogenetic evolutionary tree (NJ) of GRAS family members in *Eucalyptus grandis*. Different colors represented different branches, and groups I to XI indicated the initial classification of EgrGRAS members.

Using the neighbor joining (NJ) method for intraspecific phylogenetic analysis of *E.grandis*, the results showed that 92 EgrGRAS proteins could be categorized into 11 subgroups (Figure 2). Subgroup I comprised38 EgrGRAS members, while Subgroups II and VI each contained four members. Subgroup Ⅲ included 17 members, and Subgroups IV harbored two members. Subgroups VIII and IX each featured one member identified as Eucgr.H01257.1.v2.0 and Eucgr.A00605.1.v2.0, respectively. Subgroup V comprised 14 members, and Subgroups X and XI each contained three members.

**FIGURE 3:**
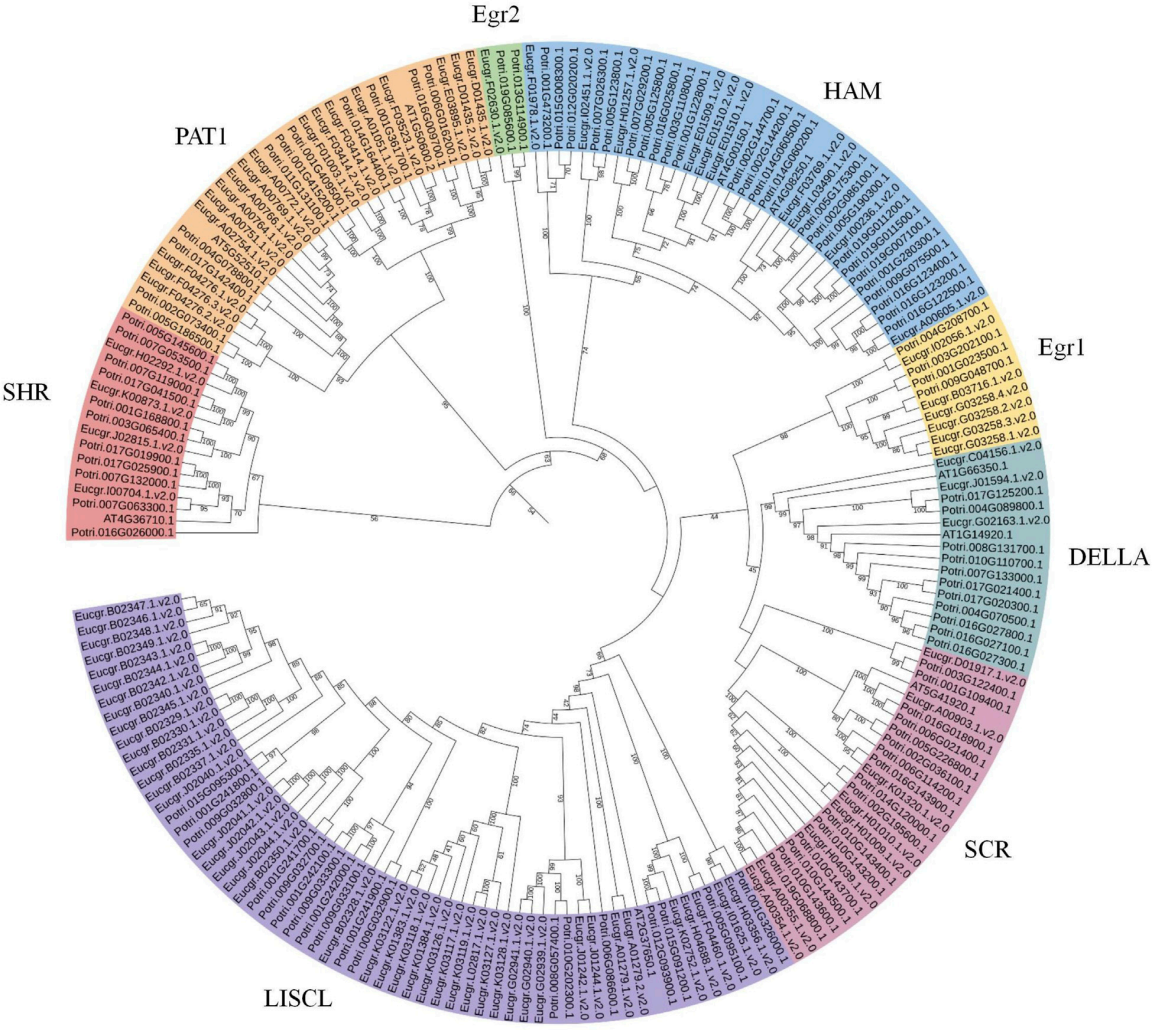
Phylogenetic analysis (ML) of GRAS proteins from *Eucalyptus grandis*, Arabidopsis and Populus. The nomenclature of the subfamilies was referenced from the subfamilies of *Arabidopsis thaliana* GRAS, with different subfamilies represented by distinct colors. Subfamilies that did not contain EgrGRAS had been removed from the figure.

To obtain more precise evolutionary information for GRAS family proteins, the GRAS protein sequences from Arabidopsis and Populus were aligned with EgrGRAS protein sequences. A phylogenetic tree for the three species was constructed using the ML method in IQ-TREE. Building upon previous research (Chen et al., 2019; Niu et al., 2017; Song et al., 2014), we named each cluster, which were categorized into eight subfamilies: LISCL, SCR, DELLA, Egr1, HAM, Egr2, PAT1, and SHR (Figure 3). The DELLA subfamily contained three EgrGRAS proteins (Eucgr.C04156.1.v2.0, Eucgr.J01594.1.v2.0, and Eucgr.G02163.1.v2.0), which were part of the EgrGRAS subgroup V in Figure 2. The LISCL subfamily included all members of subgroup I and five members of subgroup V. The remaining six members of subgroup V in Figure 2, along with four PotriGRAS members, constituted the Egr1 subfamily in Figure 3. The SCR subfamily contained all members of subgroup VII, as well as Eucgr.A00903.1.v2.0 and Eucgr.K01320.1.v2.0 from subgroup VI and Eucgr.D01917.1.v2.0 from subgroup IV. Another member of subgroup IV (Eucgr.F02630.1.v2.0) clustered with two PotriGRAS members in Figure 3, forming the subfamily Egr2. The HAM subfamily included all members of EgrGRAS subgroup VIII, IX, X, and XI, as well as Eucgr.F01978.1.v2.0 and Eucgr.I02451.1.v2.0 from subgroup VI. The PAT1 subfamily encompassed all members of subgroup III in Figure 2. All members of sugroup II in Figure 2 were assigned to the SHR subfamily in Figure 3.

### 3.3 Gene structures and motif patterns of EgrGRAS members

**FIGURE 4:**
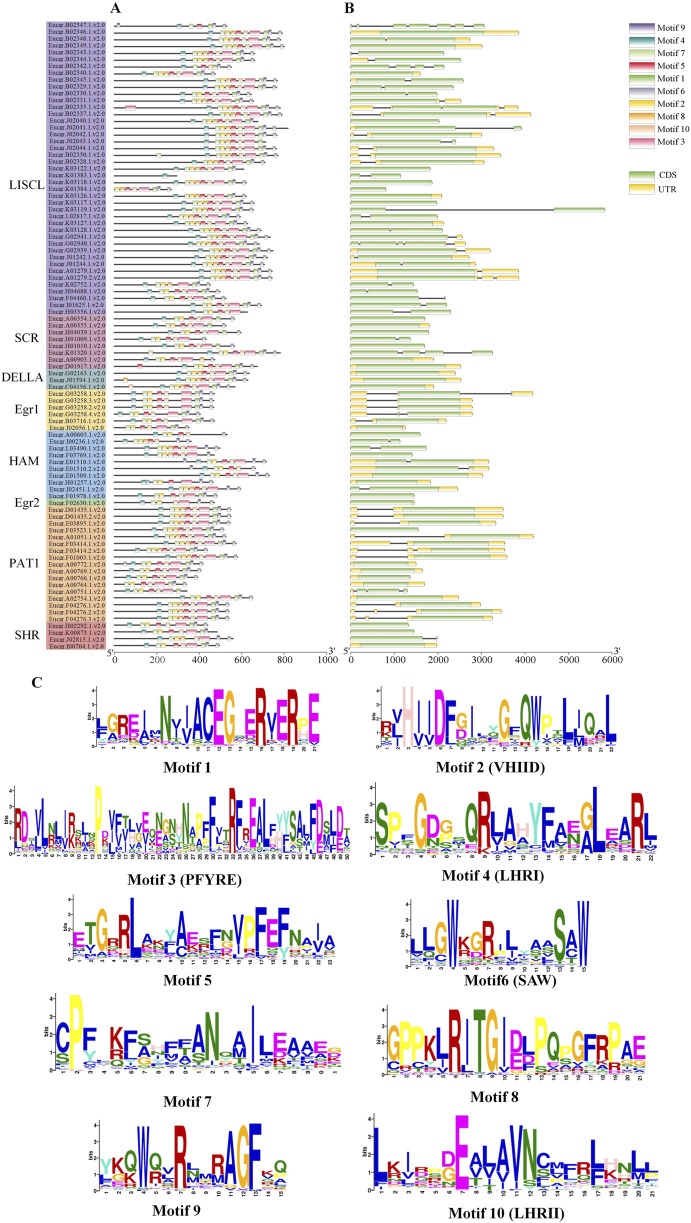
Analysis of the gene structure and conserved motif of EgrGRAS members. **(A)** Ten conserved motifs were represented with different colors. **(B)** Green indicated coding sequences (CDS), and yellow indicated untranslated regions (UTR). **(C)** The amino acid sequences of motifs and structural domain names.

The analysis of *EgrGRAS* gene structures (Figure 4B) indicated that a total of 34 *EgrGRAS* genes lacked introns (indicated by “—”), and 45 genes contained one intron. The sum of these two groups accounted for approximately 86% of *EgrGRAS* gene family members. The three *EgrGRAS* genes of the DELLA subfamily exhibited highly similar gene structures and all lack introns. The *Eucgr.B02347.1.v2.0* in the LISCL subfamily had seven exons, making it the gene with the largest number of exons in the EgrGRAS family.

The analysis of conserved protein motifs (Figure 4A) revealed that these motifs were more concentrated at the *C*-terminus compared to the *N*-terminal region. Within the same subfamily, the types and order of motifs were similar. Most members of the LISCL, DELLA, and PAT1 subfamilies contained ten motifs ranging from Motif 1 to Motif 10. The SHR subfamily was found to lack Motif 8 in Eucgr.H02292.1.v2.0, Eucgr.K00873.1.v2.0, and Eucgr.I00704.1.v2.0. Except for Eucer.F01978.1.v2.0, all members of the HAM subfamily lacked Motif 1. Most members of the SCR subfamily lacked Motif 8 and Motif 9. The amino acid composition of the identified conserved motifs was shown in Figure 4C. According to previous reports, LHRI, LHRII, VHIID, PFYRE, and SAW were the five conserved domains at the *C*-terminus of GRAS proteins (Bolle, 2004). It was discovered that in EgrGRAS, Motif 6 corresponded to the SAW domain, Motif 3 belonged to the PFYRE domain, Motif 2 was the VHIID domain, Motif 4 belonged to the LHRI domain, and Motif 10 was the LHRII domain.

### 3.4 Chromosomal distributions of *EgrGRAS* genes and synteny analysis

**FIGURE 5:**
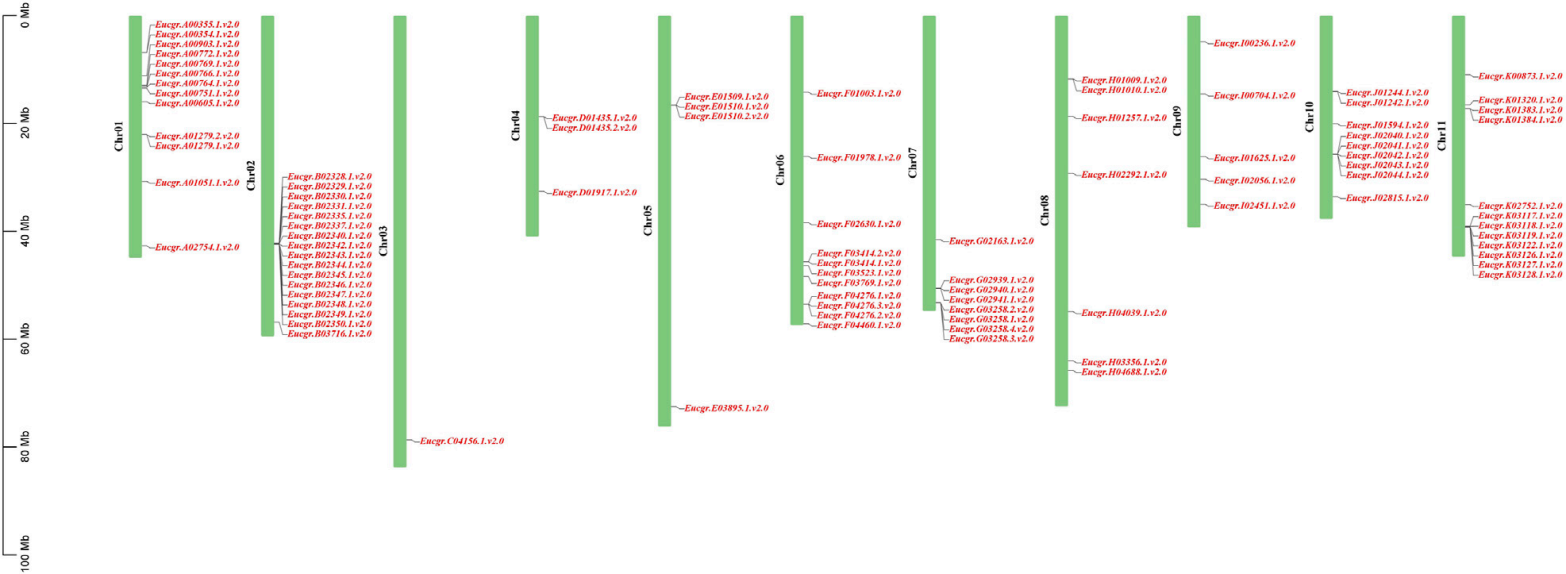
Chromosome distributions of *EgrGRAS* gene family members in 11 chromosomes.

Chromosomal location analysis revealed that a total of 90 *GRAS* genes were mapped onto the 11 chromosomes of *E. grandis* (the corresponding gene IDs are listed in Supplementary Table S1) as shown in Figure 5, with the remaining two genes (*EgrGRAS91* and *EgrGRAS92*) not yet assembled onto *E. grandis* chromosomes. Chr02 contained the most *EgrGRAS* genes and 17 *EgrGRAS* genes were located on it. The distribution of the *EgrGRAS* genes on Chr03 was the scarcest, with only one EgrGRAS31. Chr01, Chr11, Chr06 contained 13, 12, and 11 *EgrGRAS* genes, respectively; Chr10, Chr07, Chr08 harbored nine, eight, and eleven *EgrGRAS* genes, respectively; Chr09, Chr05, Chr04 possessed five, four, and three *EgrGRAS* genes, respectively.

**FIGURE 6:**
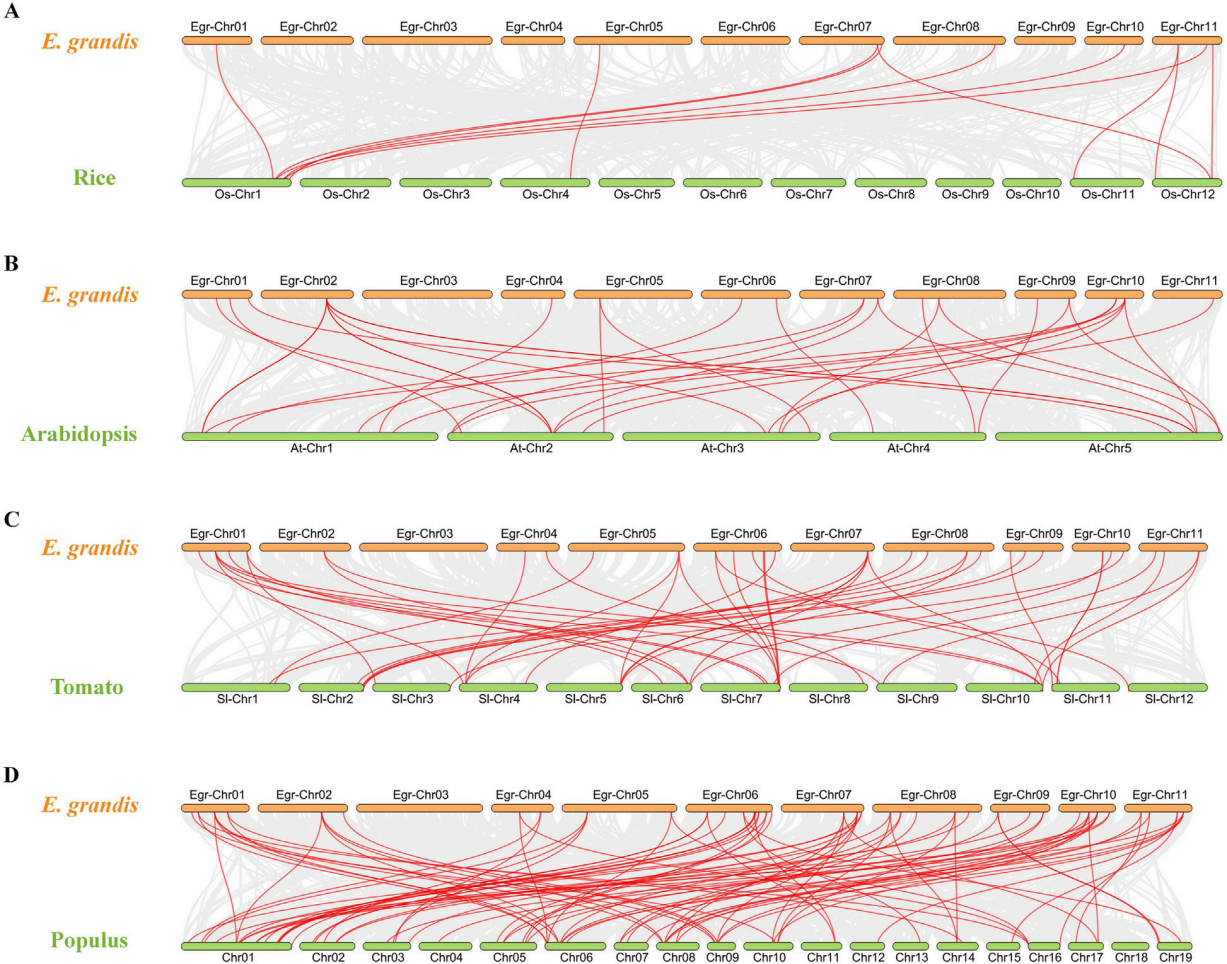
Synteny analyses of *GRAS* genes between *Eucalyptus grandis* and **(A)** rice, **(B)** Arabidopsis, **(C)** tomato, and **(D)** Populus. The syntenic blocks within the genomes of *E. grandis* and other plants were depicted with gray lines. The red lines indicated the syntenic *GRAS* gene pairs between *Eucalyptus grandis* genome and other species.

To gain a deeper understanding of the evolutionary clues for *EgrGRAS* gene family, comparative syntenic graphs (Figure 6) were constructed between *EgrGRAS* and a monocotrice), two dicots (Arabidopsis and tomato), and a woody plant (Populus). The results revealed that 42 members of *EgrGRAS* gene family showed syntenic relationships with those in rice (10), Arabidopsis (18), tomato (30), and Populus (38). The numbers of *EgrGRAS* syntenic genes in rice, Arabidopsis, tomato, and Populus were 13, 33, 49, and 83, respectively (Supplementary Table S1). Overall, a higher number of *GRAS* syntenic gene pairs were identified between *E. grandis* and dicots in comparison to monocots. Furthermore, a closer evolutionary relationship was observed between *E. grandis* and Populus.

### 3.5 The *cis*-element analysis of *EgrGRAS* genes

**FIGURE 7:**
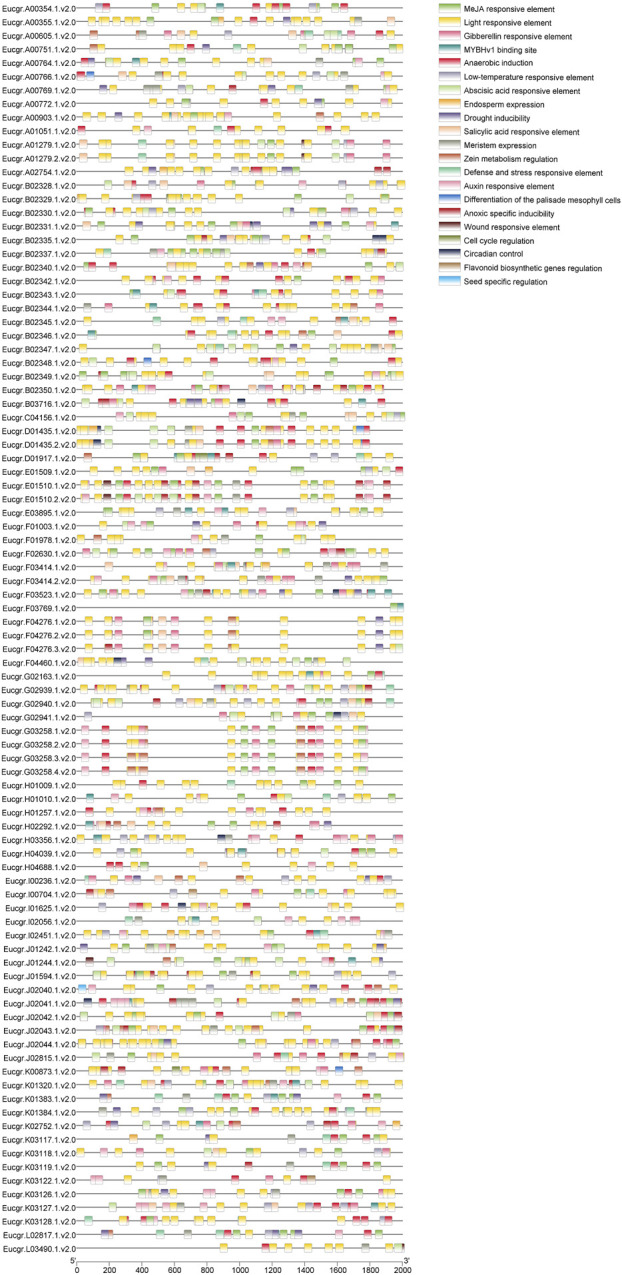
Predicted *cis*-elements in the 2,000 bp upstream of *EgrGRAS* genes. Different *cis*-elements were represented by rectangles of distinct colors.

The analysis of *cis*-acting elements (Figure 7) revealed that *EgrGRAS* genes encompassed elements associated with plant hormone responsiveness, such as salicylic acid, abscisic acid (ABA), methyl jasmonate (MeJA), auxin, and gibberellin responsive elements. Additionally, *EgrGRAS* genes included a significant number of elements related to external responses, including defense and stress responsiveness, low-temperature responsiveness, light responsiveness, drought induction, anaerobic induction, circadian control, and wound responsiveness. Among them, 76 *cis*-acting elements involved in low-temperature responsiveness were distributed in 49 *EgrGRAS* genes. Furthermore, 42 members of *EgrGRAS* gene family contained a total of 55 *cis*-acting regulatory elements related to meristem expression. A total of 47 MYBHv1 binding sites were predicted, which were presented in 33 members of the *EgrGRAS* gene family.These results suggested that *EgrGRAS* genes were involved in hormone regulation, light induction, responses to certain abiotic stresses, and plant growth.

### 3.6 Expression analysis of *GRAS* genes in leaves of diploid and triploid *Eucalyptus* and qRT-PCR analysis

**FIGURE 8:**
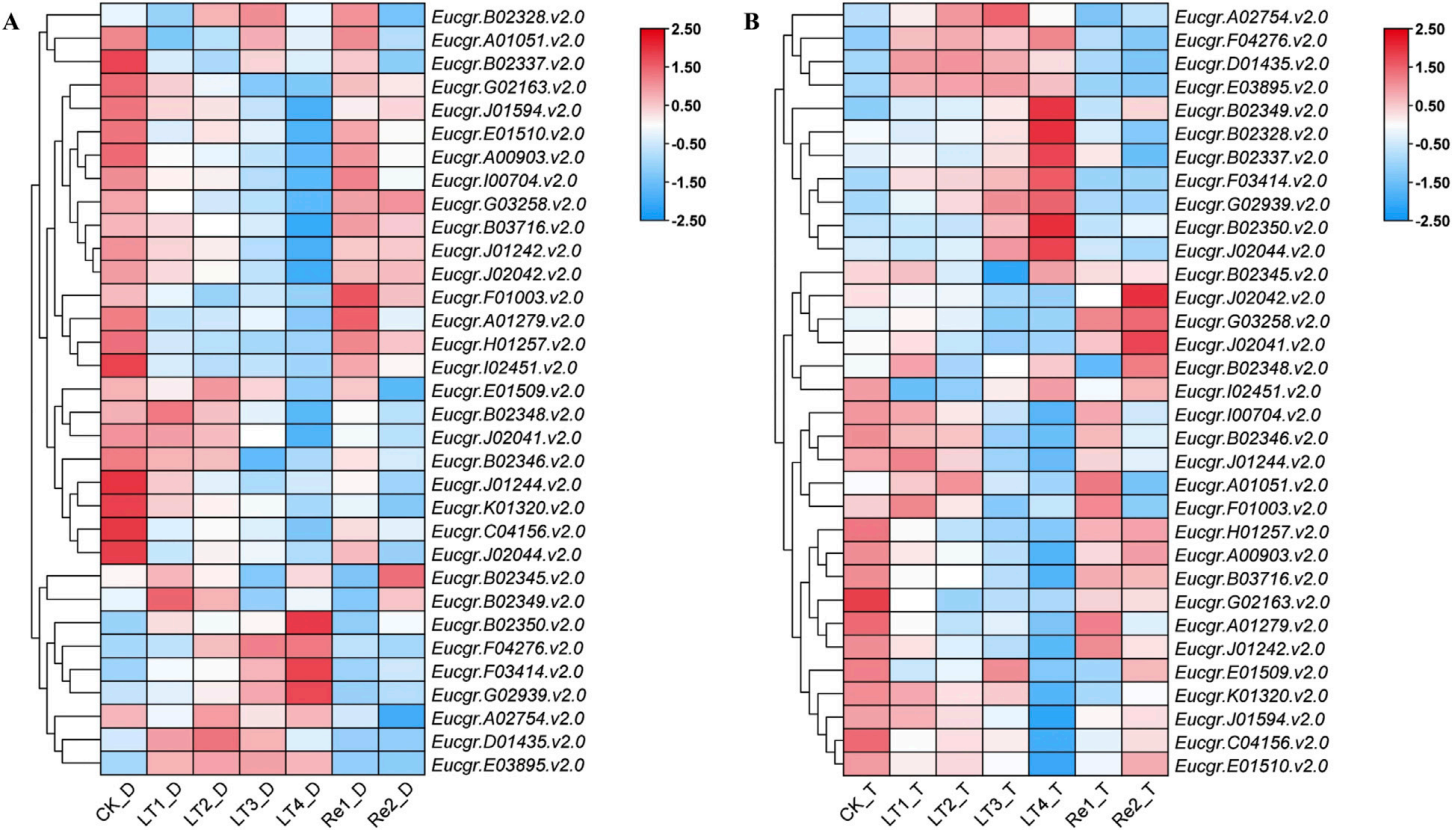
Expression heat maps of *GRAS* genes in **(A)** diploid and **(B)** triploid *Eucalyptus urophylla*. LT1, LT2, LT3, and LT4 refer to low-temperature treatments of 6, 12, 24, and 48 h, respectively. Re1 and Re2 refer to after 48 h of treatment at 4°C, the seedlings were returned to a 25°C environment for recovery 12 and 24 h, respectively. The FPKM values from RNA-seq data were transformed to Log_2_ (FPKM+1). The color scale represented the transformed values, with red indicating high expression and blue indicating low expression.

After excluding genes with extremely low expression levels and non-expressed genes across all treatments, a gene expression heatmap was constructed using the Log_2_ (FPKM+1) values of the remaining 33 GRAS genes (Figure 8). It was observed that certain *GRAS* genes exhibited low expression under the CK but high expression under other cold treatments. Genes such as *Eucgr.F04276.v2.0*, *Eucgr.F03414.v2.0*, *Eucgr.G02939.v2.0*, *Eucgr.A02754.v2.0*, *Eucgr.D01435.v2.0*, and *Eucgr.E03895.v2.0* showed similar expression patterns in in diploid and triploid *E. urophylla*, with high expression levels in at least three out of the four cold treatments (LT1 to LT4). In the diploid *E. urophylla*, the *Eucgr.B02328.v2.0* showed higher expression under LT2 and LT3 treatments, while *Eucgr.B02349.v2.0* exhibited higher expression under LT1 and LT2. However, in the triploid *E. urophylla*, these two genes displayed higher expression under LT3 and LT4. The *Eucgr.B02337*, which also showed high expression under LT3_T and LT4_T, had elevated expression under LT3_D but low expression under LT4_D. The *Eucgr.F01003.v2.0* of diploid *E*. *urophylla* exhibited low expression across all LT1 to LT4 treatments, whereas this gene in triploid *E*. *urophylla* showed high expression under LT1 and LT2. The *Eucgr.J02044.v2.0* of triploid *E*. *urophylla* exhibited high expression levels under LT3 and LT4, whereas this gene in triploid *E*. *urophylla* showed low expression levels under these same treatments.

To further validate the reliability of the transcriptomic data, we examined the expression patterns of six EgrGRAS genes using qRT-PCR. The experimental results indicated that, compared to the control group (CK), there were no significant expression differences for the majority of genes under Re1 and Re2 treatments. The *Eucgr.A02754* of diploid *E*. *urophylla* was significantly upregulated under LT2, LT3, and LT4 treatments, whereas the gene of triploid *E*. *urophylla* only exhibited upregulation under LT2. The *Eucgr.B02328* of diploid *E*. *urophylla* displayed no significant difference in expression across all experimental groups; however, the *Eucgr.B02328* of triploid *E*. *urophylla* was significantly upregulated in LT1, LT3, and LT4. The *Eucgr.B02337* showed significant upregulation under LT4_T treatment, whereas no significant expression difference was observed for this gene in LT4_D. Under LT1 and LT3, the *Eucgr.B02349* of diploid and triploid *E*. *urophylla* exhibited significant upregulation of expression; however, under LT4 treatment, this gene of diploid *E*. *urophylla* showed significant upregulation, while the gene in triploid *E*. *urophylla* demonstrated significant downregulation. The *Eucgr.F01003* of diploid *E*. *urophylla* was significantly downregulated under LT1, LT3, and LT4, whereas the gene of triploid *E*. *urophylla* was significantly upregulated under the same conditions. Similarly, we observed that the *Eucgr.J02044* of triploid *E*. *urophylla* was significantly upregulated under LT3 and LT4, while this gene of diploid *E*. *urophylla* was significantly downregulated under these treatments.

To explore the differential responses to cold stress following polyploidization in plants, the differential expression of *GRAS* genes in the leaves of triploid and diploid *Eucalyptus* was presented using TBtools, with the Log_2_ (fold change) values depicted in Figure 10. *Eucgr.F01003*.v2.0 exhibited significant upregulation in expression under CK, LT1 to LT4, and Re1, with particularly pronounced upregulation observed under LT1, LT2, and LT4. *Eucgr.B02337*.v2.0 showed significant upregulation under LT1 to LT4 and Re1, with the highest upregulation under LT4. *Eucgr.C04156*.v2.0 was significantly upregulated under LT1, LT3 and Re2. *Eucgr.A00903.1*.v2.0 was significantly downregulated under LT3 and LT4, with a more pronounced downregulation under LT4. *Eucgr.J02041*.v2.0 was significantly downregulated under LT3, whereas *Eucgr.A02754*.v2.0 was significantly upregulated under the same condition. *Eucgr.J02042*.v2.0 was significantly downregulated under CK, LT1, LT2, LT3 and Re1. *Eucgr.F03414*.v2.0 was significantly downregulated under Re1 and Re2. *Eucgr.D01435*.v2.0 was significantly downregulated under Re2, while *Eucgr.K01320*.v2.0 was significantly upregulated under the same condition. *Eucgr.B02328*.v2.0, *Eucgr.B02349*.v2.0 and *Eucgr.J02044*.v2.0 were all significantly upregulated under LT4.

## 4 Discussion

With the rapid advancement of whole-genome sequencing technologies in recent years, the genome-wide identification of *GRAS* family members has been progressively achieved across different species. The *GRAS* family represents a class of transcription factors and plays an important role in the growth, development and stress responses of plants. However, research related to *Eucalyptus GRAS* gene family is lacking. Therefore, we conducted a comprehensive bioinformatics analysis of *EgrGRAS* genes. Moreover, studies have indicated that polyploid plants typically exhibit increased tolerance to cold stress (Bon et al., 2020; Brochmann et al., 2004; Song et al., 2020). We further investigated the response patterns of *GRAS* genes to low temperatures in *E. urophylla* with different ploidy levels.

### 4.1 The evolutionary analysis and gene structure of EgrGRAS family members

In this investigation, a total of 92 *E. grandis GRAS* genes were identified. This number exceeded the count of *GRAS* genes found in Arabidopsis (32) (Tian et al., 2004), *Prunus mume* (46) (Lu et al., 2015) and rice (60) (Liu and Widmer, 2014). The number of *EgrGRAS* genes is larger, probably because its genome (430.0 Mb) is greater than those of Arabidopsis (125.0 Mb), *P. mume* (280.0 Mb), and rice (385.7 Mb). Through intraspecific evolutionary analysis, we classified EgrGRAS proteins into subgroups I to XI (Figure 2). In contrast, in the phylogenetic tree of the three species (Figure 3), 92 EgrGRAS, 9 ATGRAS, and 123 PotriGRAS clustered into eight subfamilies. The reason for the different clustering results is the use of different analytical methods. Referring to the classification of the Arabidopsis GRAS family proteins (Liu and Widmer, 2014; Tian et al., 2004), six clusters within the phylogenetic tree were named as LISCL, SCR, DELLA, HAM, PAT1, and SHR. The remaining two clusters were denoted as Egr1 and Egr2, which did not contain ATGRAS but only PotriGRAS and EgrGRAS. This might represent that these subfamilies were specific evolutionary branches unique to woody dicots. The results of the synteny analysis (Figure 6) revealed that 42 *EgrGRAS* genes had 178 syntenic *GRAS* genes on the chromosomes of rice, Arabidopsis, tomato, and Populus. Notably, the LISCL subfamily members *Eucgr.A01279.1.v2.0*, *Eucgr.B02328.1.v2.0*, *Eucgr.K03117.1.v2.0*, *Eucgr.G02939.1.v2.0*, and *Eucgr.J02040.1.v2.0*, as well as the SCR subfamily member *Eucgr.K01320.1.v2.0* and the HAM subfamily member *Eucgr.E01509.1.v2.0*, all have syntenic gene pairs with the other four species. This indicates that these seven *EgrGRAS* genes are highly conserved and may have originated prior to the divergence of these species.

Chromosomal localization of *EgrGRAS* members was performed, and the results showed an uneven distribution among the 90 members that were mapped to chromosomes (Figure 5). Approximately 46% of the *EgrGRAS* genes were distributed on chr01, chr02, and chr11, forming short clusters of tandem repeats. In contrast, chr03 harbored only one *EgrGRAS* gene. Upon analyzing the gene structure of all *EgrGRAS* members, we observed that 86% of *EgrGRAS* family members lack introns or contain only one intron (Figure 4B). This was similar to the findings in *Arabidopsis*, tomato, and soybean (*Glycine max*), where *GRAS* genes lacking introns accounted for 67.6% (Tian et al., 2004), 77.4% (Niu et al., 2017), and 77.8% (Wang et al., 2020) respectively. Genes within the same subfamily exhibited a similar exon-intron distribution pattern. Some genes within the LISCL, SCR, and PAT1 subfamilies contained a higher number of introns, which may indicate that they play significant roles in the evolutionary process of *Eucalyptus*.

### 4.2 Analysis of the physicochemical properties and protein motifs of EgrGRAS transcription factors

In this study, the amino acid quantity, molecular weight and pI of proteins encoded by members of *EgrGRAS* gene family ranged from 267 to 817aa, 31.5–92.8 kDa and 4.86 to 9.15, respectively (Supplementary Table S1). These findings were basically consistent with the results from studies on *GRAS* genes encoding proteins in soybean (Wang et al., 2020) and a wild relative of sweet potato (*Ipomoea trifida*) (Chen et al., 2019), indicating that GRAS proteins from different species shared similar physicochemical properties. We cloned an *EgrGRAS* gene, and transient transformation studies revealed its localization to the cell nucleus, which is consistent with the result of subcellular localization predictions. More than half of the EgrGRAS proteins are localized within the nucleus, suggesting that they may be important nuclear transcription factors.

Conserved motif analysis was conducted on 92 EgrGRAS proteins and proteins from the same subfamily exhibited similar motif structures and arrangements (Figure 4A), indicating potential functional similarities among EgrGRAS proteins. The vast majority of EgrGRAS proteins contained Motif6, Motif3, Motif2, Motif4, and Motif10, which were respectively located within the SAW, PFYRE, VHIID, LHRI, and LHRII domains. Studies have indicated that these motifs can mediate protein-protein and protein-DNA interactions. Most members of the LISCL, DELLA, and PAT1 subfamilies possessed Motif1, Motif8, and Motif9, while some members of other subfamilies lacked these motifs. This suggests that proteins within the LISCL, DELLA, and PAT1 subfamilies may exert unique functions.

### 4.3 Analysis of *cis*-acting elements and expression of *EgrGRAS* genes in *Eucalyptus urophylla* with different ploidy under cold stress

Analysis of the *cis*-acting elements of *EgrGRAS* genes had been revealed that the promoter region predominantly contained response elements for hormones, light, and stress (Figure 7). Among these, the stress responses included abiotic stresses such as low temperature and drought. Studies have indicated that plant hormones play a significant role in the regulation of signal transduction pathways and in response to external stresses (Peleg and Blumwald, 2011), including ABA (Huang et al., 2012) and MeJA (Yuan et al., 2017). This study found that the promoter region of *EgrGRAS* genes contained response elements for hormones such as ABA and MeJA, suggesting that *GRAS* genes have a certain function in the regulatory network of eucalyptus in response to external stresses. Over half of *EgrGRAS* genes contained low-temperature responsive elements, specifically, 28 members from the LISCL subfamily, 5 members from the SCR subfamily, 2 members (*Eucgr.C04156.1.v2.0* and *Eucgr.J01594.1.v2.0*) from the DELLA subfamily, 4 members (*Eucgr.A00605.1.v2.0*, *Eucgr.I00236.1.v2.0*, *Eucgr.E01509.1.v2.0*,and *Eucgr.I02451.1.v2.0*) from the HAM subfamily, 5 members from the PAT1 subfamily, and 3 members (*Eucgr.K00873.1.v2.0*, *Eucgr.J02815.1.v2.0*,and *Eucgr.I00704.1.v2.0*) from the SHR subfamily. These *GRAS* genes may play an important role in cold resistance. Studies on the function of *GRAS* genes in cotton and tea plant (*Camellia sinensis*) have also identified the capability of *GRAS* genes to respond to cold stress (Wang et al., 2018; Zhang et al., 2018).

**FIGURE 9:**
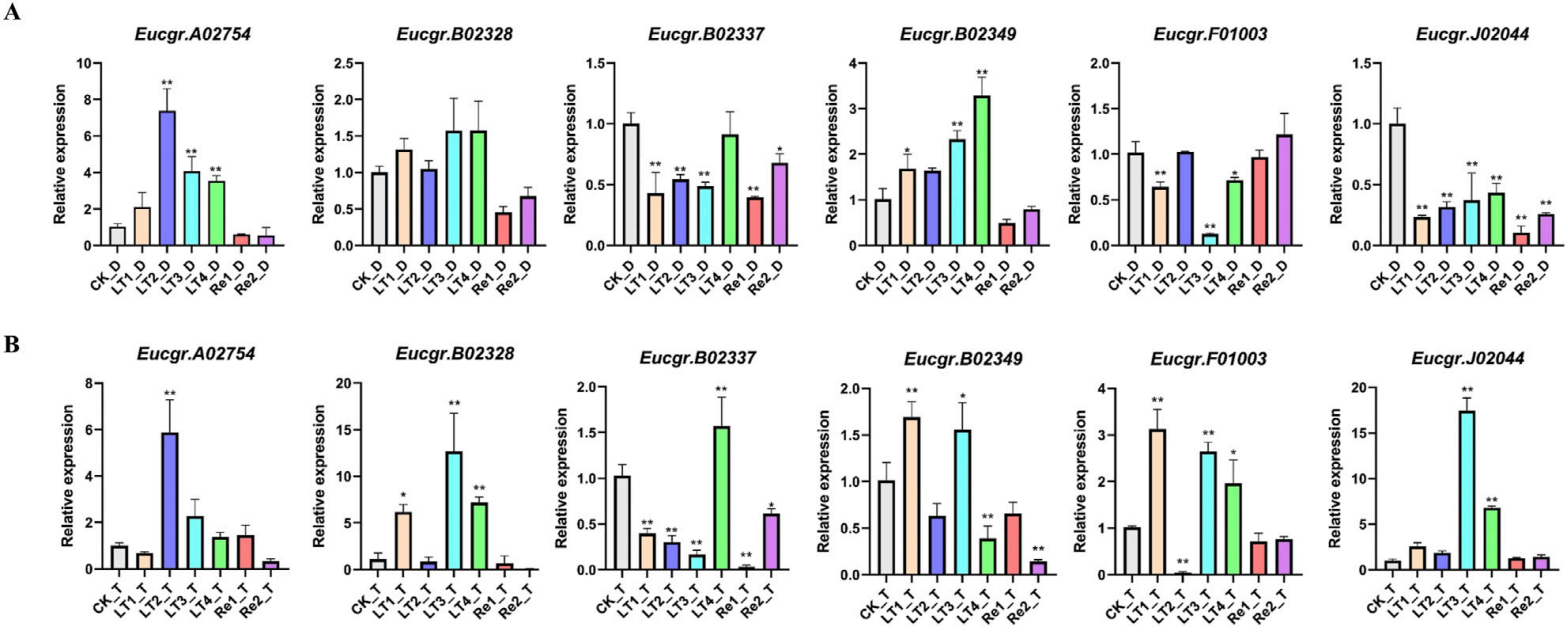
The expression patterns of GRAS genes in **(A)** diploid and **(B)** triploid *Eucalyptus urophylla* leaves by qRT-PCR analysis. The *y*-axis represented the relative expression levels of *EgrGRAS*, while the *x*-axis denoted the control group and various experimental groups. Standard error bars indicated the standard deviation of three replicates. Asterisks signified significant upregulation or downregulation of *EgrGRAS* genes in other treatment groups compared to the CK (*p < 0.05, **p < 0.01).

The transcriptome data analysis of *GRAS* genes in the leaves of *E. urophylla* with different ploidy levels identified 33 genes of particular interest (Figure 8). qRT-PCR experiments were conducted to examine the expression of six of these genes (Figure 9). Within the same subfamily, multiple genes exhibited similar expression profiles. *Eucgr.A02754.v2.0*, a member of the PAT1 subfamily, was significantly upregulated under LT2_D, LT3_D, and LT4_D treatments (Figures 8A, 9A). Genes of the same subfamily, *Eucgr.D01435.v2.0*, *Eucgr.E03895.v2.0*, and *Eucgr.F04276.v2.0*, exhibited similar expression patterns, showing significant upregulation in at least three out of the four treatments (LT1 to LT4) in both diploid and triploid *E. urophylla* (Figure 8). *Eucgr.B02328.v2.0*, belonging to the LISCL subfamily, was significantly upregulated under LT3_T and LT4_T (Figures 8B, 9B). Other genes of this subfamily, including *Eucgr.B02346.v2.0*, *Eucgr.B02348.v2.0*, *Eucgr.B02349.v2.0*, *Eucgr.B02345.v2.0*, *Eucgr.B02350.v2.0*, and *Eucgr.G02939.v2.0*, displayed significant upregulation in at least two out of the four treatments (LT1 to LT4) in both diploid and triploid *E. urophylla* (Figure 8). The study also found that the expression patterns of the same gene varied between diploid and triploid *E. urophylla*. Both transcriptome analysis and qRT-PCR experiments revealed that, compared to CK, *Eucgr.J02044.v2.0* was significantly downregulated under LT3_D and LT4_D but upregulated under LT3_T and LT4_T.

**FIGURE 10:**
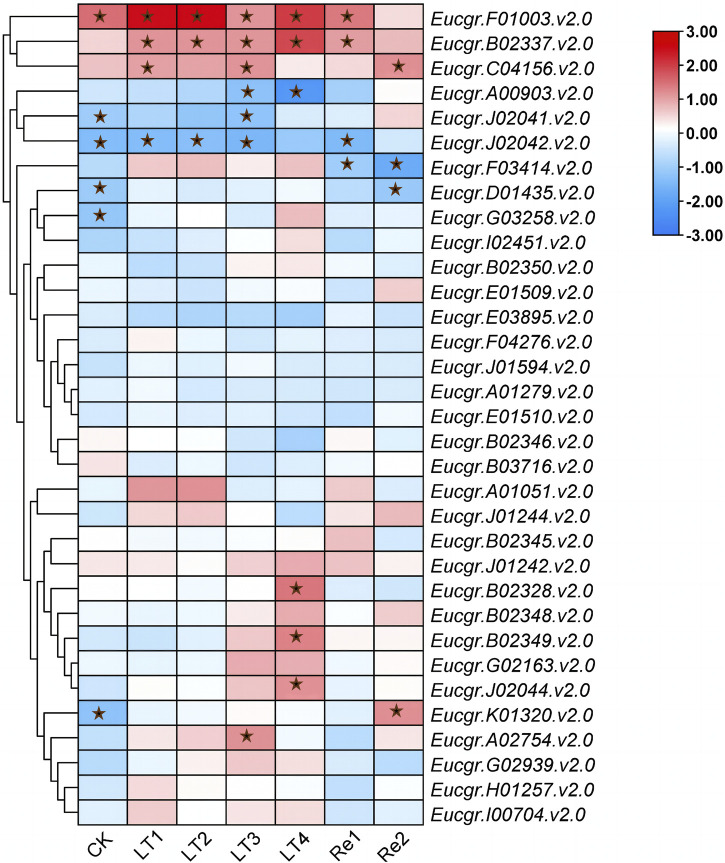
Differential expression of *GRAS* genes in triploid and diploid *Eucalyptus urophylla* under different cold treatment durations and recovery periods. The heatmap presented Log_2_ (fold change) values derived from transcriptomic data, where red indicated upregulated expression and blue indicated downregulated expression, with asterisks indicating modules that were significantly differentially expressed (*p* < 0.05).

A study about Castor Beans has shown that genes from the LISCL and PAT1 subfamilies responded to various environmental stresses, including cold stress (Xu et al., 2016). Previous research on the overexpression of the VaPAT1 transcription factor in Arabidopsis has confirmed the important role of PAT1 subfamily genes in enhancing plant tolerance to abiotic stresses (Yuan et al., 2016). Many studies have indicated that the DELLA subfamily is involved in the response to cold stress and other abiotic stresses (Achard and Genschik, 2009; Achard et al., 2008). Using the expression levels of diploids as a reference, the differential expression in triploids was depicted in Figure 10. *Eucgr.B02328*.v2.0, *Eucgr.B02337*.v2.0, and *Eucgr.J02044*.v2.0 from the LISCL subfamily were all significantly upregulated under LT4 treatment. The *Eucgr.F01003.1*.v2.0 from the PAT1 subfamily was significantly upregulated under LT1, LT2, and LT4. Comparing the relative expression levels of these four genes in Figures 9A, B also yielded this result. Additionally, we found that the*Eucgr.C04156*.v2.0 from the DELLA subfamily exhibited significant upregulation under LT1, LT3 and Re2 (Figure 10). Comparing the expression patterns of this gene in Figures 8A, B also confirmed this result. Recent research has found that changes in gene expression in polyploid plants, particularly the upregulation of cold-resistant gene expression, may be a significant reason for their enhanced cold tolerance (Bon et al., 2020; Song et al., 2020; Tossi et al., 2022). In our study, it was evident that *Eucgr.C04156*.v2.0, *Eucgr.B02328*.v2.0, *Eucgr.B02337*.v2.0, *Eucgr.J02044*.v2.0, and *Eucgr.F01003*.v2.0 were likely key genes in the response to cold stress in triploid *Eucalyptus*.

## 5 Conclusion

In summary, this study has identified 92 *EgrGRAS* genes, of which 90 were unevenly distributed across 11 chromosomes. Based on the phylogenetic tree constructed with Arabidopsis and Populus, the 92 *GRAS* genes were classified into eight subfamilies. Synteny analysis revealed that *E. grandis* GRAS shared more syntenic gene pairs with Populus *GRAS*, indicating a closer phylogenetic relationship between the two species. Gene structure analysis found that most *EgrGRAS* genes lacked introns and had similar intron-exon structures. Physicochemical property analysis showed that the physicochemical properties of EgrGRAS proteins were similar to those of GRAS proteins from other species. Subcellular localization prediction suggested that most EgrGRAS proteins were localized to the nucleus. Tobacco transient transformation experiments further confirmed that GRAS proteins are nuclear transcription factors. Conserved motif analysis found that GRAS proteins from the same subfamily had similar motif composition and order, while different subfamilies exhibited differences. *C*is-acting element analysis revealed that *EgrGRAS* contained numerous *cis*-acting elements associated with plant growth and response to light induction, hormones, and stress. Transcriptome analysis and qRT-PCR experiments revealed that certain members of the DELLA, LISCL and PAT1 subfamilies were highly expressed under cold stress in both diploid and triploid *E*. *urophylla*, with significantly upregulated expression in triploid plants compared to diploid plants. These genes may play an extremely important role in the response of *Eucalyptus* to cold stress. This study laid the foundation for future in-depth research on the function of *Eucalyptus GRAS* genes and the regulatory mechanisms of triploid *Eucalyptus* response to low-temperature stress.

## Data Availability

All relevant data is contained within the article: The original contributions presented in the study are included in the article/Supplementary Material, further inquiries can be directed to the corresponding authors.

## References

[B1] Achard P. Genschik P. (2009). Releasing the brakes of plant growth: how GAs shutdown DELLA proteins. J. Exp. Bot. 60 (4), 1085–1092. 10.1093/jxb/ern301 19043067

[B2] Achard P. Gong F. Cheminant S. Alioua M. Hedden P. Genschik P. (2008). The cold-inducible CBF1 factor-dependent signaling pathway modulates the accumulation of the growth-repressing DELLA proteins via its effect on gibberellin metabolism. Plant cell 20 (8), 2117–2129. 10.1105/tpc.108.058941 18757556 PMC2553604

[B3] Bailey T. L. Johnson J. Grant C. E. Noble W. S. (2015). The MEME suite. Nucleic. acids. Res. 43 (W1), W39–W49. 10.1093/nar/gkv416 25953851 PMC4489269

[B4] Blum M. Chang H. Chuguransky S. Grego T. Kandasaamy S. Mitchell A. (2021). The InterPro protein families and domains database: 20 years on. Nucleic. acids. Res. 49 (D1), D344–D354. 10.1093/nar/gkaa977 33156333 PMC7778928

[B5] Bolle C. (2004). The role of GRAS proteins in plant signal transduction and development. Planta 218 (5), 683–692. 10.1007/s00425-004-1203-z 14760535

[B6] Bolle C. Koncz C. Chua N. H. (2000). PAT1, a new member of the GRAS family, is involved in phytochrome a signal transduction. Genes. Dev. 14 (10), 1269–1278. 10.1101/gad.14.10.1269 10817761 PMC316623

[B7] Bon P. V. Harwood C. E. Nghiem Q. C. Thinh H. H. Son D. H. Chinh N. V. (2020). Growth of triploid and diploidacacia clones in three contrasting environments in Viet Nam. Aust. For. 83 (4), 265–274. 10.1080/00049158.2020.1819009

[B8] Brochmann C. Brysting A. K. Alsos I. G. Borgen L. Grundt H. H. Scheen A. C. (2004). Polyploidy in arctic plants. Biol. J. Linn. Soc. 82(4), 521–536. 10.1111/j.1095-8312.2004.00337.x

[B56] Capella-Gutierrez S. Silla-Martinez J. M. Gabaldon T. (2009). Trimal: a tool for automated alignment trimming in large-scale phylogenetic analyses. Bioinformatics 25(15), 1972–1973. 10.1093/bioinformatics/btp348 19505945 PMC2712344

[B9] Cassan-Wang H. Soler M. Yu H. Camargo E. L. Carocha V. Ladouce N. (2012). Reference genes for high-throughput quantitative reverse transcription-PCR analysis of gene expression in organs and tissues of Eucalyptus grown in various environmental conditions. Plant Cell Physiol. 53 (12), 2101–2116. 10.1093/pcp/pcs152 23161857

[B10] Chen C. Chen H. Zhang Y. Thomas H. R. Frank M. H. He Y. (2020). TBtools: an integrative toolkit developed for interactive analyses of big biological data. Mol. Plant. 13 (8), 1194–1202. 10.1016/j.molp.2020.06.009 32585190

[B11] Chen H. Li J. Qiu B. Zhao Y. Liu Z. Yang J. (2021). Long non-coding RNA and its regulatory network response to cold stress in eucalyptus urophylla s.t.blake. Forests 12 (7), 836. 10.3390/f12070836

[B12] Chen Y. Zhu P. Wu S. Lu Y. Sun J. Cao Q. (2019). Identification and expression analysis of GRAS transcription factors in the wild relative of sweet potato ipomoea trifida. BMC Genomics 20 (1), 911. 10.1186/s12864-019-6316-7 31783728 PMC6884806

[B13] Czikkel B. E. Maxwell D. P. (2007). NtGRAS1, a novel stress-induced member of the GRAS family in tobacco, localizes to the nucleus. J. Plant Physiol. 164 (9), 1220–1230. 10.1016/j.jplph.2006.07.010 17007961

[B14] Di Laurenzio L. Wysocka-Diller J. Malamy J. E. Pysh L. Helariutta Y. Freshour G. (1996). The SCARECROW gene regulates an asymmetric cell division that is essential for generating the radial organization of the arabidopsis root. Cell 86 (3), 423–433. 10.1016/s0092-8674(00)80115-4 8756724

[B15] Edgar R. C. (2004). MUSCLE: multiple sequence alignment with high accuracy and high throughput. Nucleic. acids. Res. 32 (5), 1792–1797. 10.1093/nar/gkh340 15034147 PMC390337

[B16] Helariutta Y. Fukaki H. Wysocka-Diller J. Nakajima K. Jung J. Sena G. (2000). The SHORT-ROOT gene controls radial patterning of the arabidopsis root through radial signaling. Cell. 101 (5), 555–567. 10.1016/s0092-8674(00)80865-x 10850497

[B17] Hirano K. Asano K. Tsuji H. Kawamura M. Mori H. Kitano H. (2010). Characterization of the molecular mechanism underlying gibberellin perception complex formation in rice. Plant. Cell. 22 (8), 2680–2696. 10.1105/tpc.110.075549 20716699 PMC2947161

[B18] Hirsch S. Oldroyd G. E. (2009). GRAS-domain transcription factors that regulate plant development. Plant Signal. Behav. 4 (8), 698–700. 10.4161/psb.4.8.9176 19820314 PMC2801379

[B19] Huang G. T. Ma S. L. Bai L. P. Zhang L. Ma H. Jia P. (2012). Signal transduction during cold, salt, and drought stresses in plants. Mol. Biol. Rep. 39 (2), 969–987. 10.1007/s11033-011-0823-1 21573796

[B20] Jin J. Tian F. Yang D. C. Meng Y. Q. Kong L. Luo J. (2017). PlantTFDB 4.0: toward a central hub for transcription factors and regulatory interactions in plants. Nucleic. acids. Res. 45 (D1), D1040-D1045–D1045. 10.1093/nar/gkw982 27924042 PMC5210657

[B21] Lamesch P. Berardini T. Z. Li D. Swarbreck D. Wilks C. Sasidharan R. (2012). The arabidopsis information resource (TAIR): improved gene annotation and new tools. Nucleic. acids. Res. 40, D1202–D1210. 10.1093/nar/gkr1090 22140109 PMC3245047

[B22] Lee M. H. Kim B. Song S. K. Heo J. O. Yu N. I. Lee S. A. (2008). Large-scale analysis of the GRAS gene family in arabidopsis thaliana. Plant Mol. Biol. 67 (6), 659–670. 10.1007/s11103-008-9345-1 18500650

[B23] Lescot M. Dehais P. Thijs G. Marchal K. Moreau Y. Van de Peer Y. (2002). PlantCARE, a database of plant cis-acting regulatory elements and a portal to tools for *in silico* analysis of promoter sequences. Nucleic. acids. Res. 30 (1), 325–327. 10.1093/nar/30.1.325 11752327 PMC99092

[B24] Letunic I. Bork P. (2024). Interactive tree of life (iTOL) v3: an online tool for the display and annotation of phylogenetic and other trees. Nucleic. acids. Res. 44, W242–W245. 10.1093/nar/gkw290 PMC4987883 27095192

[B25] Letunic I. Khedkar S. Bork P. (2021). SMART: recent updates, new developments and status in 2020. Nucleic. acids. Res. 49 (D1), D458–D460. 10.1093/nar/gkaa937 33104802 PMC7778883

[B26] Lin Q. Wang D. Dong H. Gu S. Cheng Z. Gong J. (2012). Rice APC/c(TE) controls tillering by mediating the degradation of MONOCULM 1. Nat. Commun. 3, 752. 10.1038/ncomms1716 22434195 PMC3316886

[B27] Liu X. Widmer A. (2014). Genome-wide comparative analysis of the GRAS gene family in populus, arabidopsis and rice. Plant Mol. Biol. Rep. 32 (6), 1129–1145. 10.1007/s11105-014-0721-5

[B28] Livak K. J. Schmittgen T. D. (2001). Analysis of relative gene expression data using real-time quantitative PCR and the 2(-delta delta c(t)) method. Methods 25 (4), 402–408. 10.1006/meth.2001.1262 11846609

[B29] Lu J. Wang T. Xu Z. Sun L. Zhang Q. (2015). Genome-wide analysis of the GRAS gene family in prunus mume. Mol. Genet. Genomics. 290 (1), 303–317. 10.1007/s00438-014-0918-1 25245166

[B30] Lu S. Wang J. Chitsaz F. Derbyshire M. K. Geer R. C. Gonzales N. R. (2020). CDD/SPARCLE: the conserved domain database in 2020. Nucleic. acids. Res. 48 (D1), D265-D268–D268. 10.1093/nar/gkz991 31777944 PMC6943070

[B31] Minh B. Q. Schmidt H. A. Chernomor O. Schrempf D. Woodhams M. D. von Haeseler A. (2020). IQ-TREE 2: new models and efficient methods for phylogenetic inference in the genomic era. Mol. Biol. Evol. 37 (5), 1530–1534. 10.1093/molbev/msaa015 32011700 PMC7182206

[B32] Niu Y. Zhao T. Xu X. Li J. (2017). Genome-wide identification and characterization of GRAS transcription factors in tomato (solanum lycopersicum). PeerJ 5, e3955. 10.7717/peerj.3955 29134140 PMC5681854

[B33] Peleg Z. Blumwald E. (2011). Hormone balance and abiotic stress tolerance in crop plants. Curr. Opin. Plant Biol. 14 (3), 290–295. 10.1016/j.pbi.2011.02.001 21377404

[B34] Peng J. Carol P. Richards D. E. King K. E. Cowling R. J. Murphy G. P. (1997). The arabidopsis GAI gene defines a signaling pathway that negatively regulates gibberellin responses. Genes. Dev. 11 (23), 3194–3205. 10.1101/gad.11.23.3194 9389651 PMC316750

[B35] Pysh L. D. Diller J. W. W. Camilleri C. Bouchez D. Benfey P. N. (1999). The GRAS gene family in arabidopsis: sequence characterization and basic expression analysis of the SCARECROW‐LIKE genes. Plant J. 18 (1), 111–119. 10.1046/j.1365-313X.1999.00431.x 10341448

[B36] Silverstone A. L. Ciampaglio C. N. Sun T. (1998). The arabidopsis RGA gene encodes a transcriptional regulator repressing the gibberellin signal transduction pathway. Plant. Cell 10 (2), 155–169. 10.1105/tpc.10.2.155 9490740 PMC143987

[B37] Song X. Liu T. Duan W. Ma Q. Ren J. Wang Z. (2014). Genome-wide analysis of the GRAS gene family in Chinese cabbage (brassica rapa ssp. Pekinensis). Genomics 103 (1), 135–146. 10.1016/j.ygeno.2013.12.004 24365788

[B38] Song X. Wang J. Sun P. Ma X. Yang Q. Hu J. (2020). Preferential gene retention increases the robustness of cold regulation in brassicaceae and other plants after polyploidization. Hortic. Res. 7 (1), 20. 10.1038/s41438-020-0253-0 32133148 PMC7035258

[B39] Sun X. Jones W. T. Rikkerink E. H. (2012). GRAS proteins: the versatile roles of intrinsically disordered proteins in plant signalling. Biochem. J. 442 (1), 1–12. 10.1042/BJ20111766 22280012

[B40] Tamura K. Stecher G. Kumar S. (2021). MEGA11: molecular evolutionary genetics analysis version 11. Mol. Biol. Evol. 38 (7), 3022–3027. 10.1093/molbev/msab120 33892491 PMC8233496

[B41] Tanabe S. Onodera H. Hara N. Ishii-Minami N. Day B. Fujisawa Y. (2016). The elicitor-responsive gene for a GRAS family protein, CIGR2, suppresses cell death in rice inoculated with rice blast fungus via activation of a heat shock transcription factor, OsHsf23. Biosci. Biotechnol. Biochem. 80 (1), 145–151. 10.1080/09168451.2015.1075866 26287768

[B42] Tian C. Wan P. Sun S. Li J. Chen M. (2004). Genome-wide analysis of the GRAS gene family in rice and arabidopsis. Plant Mol. Biol. 54 (4), 519–532. 10.1023/B:PLAN.0000038256.89809.57 15316287

[B43] Tong N. Li D. Zhang S. Tang M. Chen Y. Zhang Z. (2023). Genome-wide identification and expression analysis of the GRAS family under low-temperature stress in bananas. Front. Plant Sci. 14, 1216070. 10.3389/fpls.2023.1216070 37719217 PMC10502232

[B44] Tossi V. E. Martinez Tosar L. J. Laino L. E. Iannicelli J. Regalado J. J. Escandon A. S. (2022). Impact of polyploidy on plant tolerance to abiotic and biotic stresses. Front. Plant Sci. 13, 869423. 10.3389/fpls.2022.869423 36072313 PMC9441891

[B45] Vilasboa J. Da Costa C. T. Fett-Neto A. G. (2019). Rooting of eucalypt cuttings as a problem-solving oriented model in plant biology. Prog. Biophysics Mol. Biol. An Int. Rev. J. 146, 85–97. 10.1016/j.pbiomolbio.2018.12.007 30557533

[B46] Wang L. Ding X. Gao Y. Yang S. (2020). Genome-wide identification and characterization of GRAS genes in soybean (glycine max). BMC Plant Biol. 20 (1), 415. 10.1186/s12870-020-02636-5 32891114 PMC7487615

[B47] Wang Y. Tang H. Debarry J. D. Tan X. Li J. Wang X. (2012). MCScanX: a toolkit for detection and evolutionary analysis of gene synteny and collinearity. Nucleic. acids. Res. 40 (7), e49. 10.1093/nar/gkr1293 22217600 PMC3326336

[B48] Wang Y. X. Liu Z. W. Wu Z. J. Li H. Wang W. L. Cui X. (2018). Genome-wide identification and expression analysis of GRAS family transcription factors in tea plant (Camellia sinensis). Sci. Rep. 8 (1), 3949. 10.1038/s41598-018-22275-z 29500448 PMC5834537

[B49] Wang Z. Wong D. C. J. Wang Y. Xu G. Ren C. Liu Y. (2021). GRAS-domain transcription factor PAT1 regulates jasmonic acid biosynthesis in grape cold stress response. Plant physiol. (Bethesda). 186 (3), 1660–1678. 10.1093/plphys/kiab142 PMC8260143 33752238

[B50] Xu W. Chen Z. Ahmed N. Han B. Cui Q. Liu A. (2016). Genome-wide identification, evolutionary analysis, and stress responses of the GRAS gene family in castor beans. Int. J. Mol. Sci. 17 (7), 1004. 10.3390/ijms17071004 27347937 PMC4964380

[B51] Yang J. Wang J. Liu Z. Xiong T. Lan J. Han Q. (2018). Megaspore chromosome doubling in eucalyptus urophylla s.t. Blake induced by colchicine treatment to produce triploids. Forests 9 (11), 728. 10.3390/f9110728

[B52] Yuan H. M. Sheng Y. Chen W. J. Lu Y. Q. Tang X. Ou-Yang M. (2017). Overexpression of hevea brasiliensis HbICE1 enhances cold tolerance in arabidopsis. Front. Plant Sci. 8, 1462. 10.3389/fpls.2017.01462 28878797 PMC5572258

[B53] Yuan Y. Fang L. Karungo S. K. Zhang L. Gao Y. Li S. (2016). Overexpression of VaPAT1, a GRAS transcription factor from Vitis amurensis, confers abiotic stress tolerance in Arabidopsis. Plant Cell Rep. 35 (3), 655–666. 10.1007/s00299-015-1910-x 26687967

[B54] Zhang B. Liu J. Yang Z. E. Chen E. Y. Zhang C. J. Zhang X. Y. (2018). Genome-wide analysis of GRAS transcription factor gene family in gossypium hirsutum l. BMC Genomics 19, 348. 10.1186/s12864-018-4722-x 29743013 PMC5944045

[B55] Zhang Y. Wang X. (2021). Geographical spatial distribution and productivity dynamic change of eucalyptus plantations in China. Sci. Rep. 11 (1), 19764. 10.1038/s41598-021-97089-7 34611175 PMC8492638

